# Super-Resolution Molecular and Functional Imaging of Nanoscale Architectures in Life and Materials Science

**DOI:** 10.3389/fbioe.2014.00020

**Published:** 2014-06-12

**Authors:** Satoshi Habuchi

**Affiliations:** ^1^Biological and Environmental Sciences and Engineering Division, King Abdullah University of Science and Technology, Jeddah, Saudi Arabia

**Keywords:** super-resolution, fluorescence microscopy, nanostructures, cellular imaging, nanomaterials

## Abstract

Super-resolution (SR) fluorescence microscopy has been revolutionizing the way in which we investigate the structures, dynamics, and functions of a wide range of nanoscale systems. In this review, I describe the current state of various SR fluorescence microscopy techniques along with the latest developments of fluorophores and labeling for the SR microscopy. I discuss the applications of SR microscopy in the fields of life science and materials science with a special emphasis on quantitative molecular imaging and nanoscale functional imaging. These studies open new opportunities for unraveling the physical, chemical, and optical properties of a wide range of nanoscale architectures together with their nanostructures and will enable the development of new (bio-)nanotechnology.

## Introduction

The revealing of microscopic structures and their associated dynamics comprise essential elements of modern research studies in biological as well as materials science. NMR spectroscopy and X-ray crystallography are the primary tools for characterizing structure and dynamics at the molecular level. Electron microscopy (EM) has been recognized as a versatile means by which to investigate nanoscale structures of biological specimens, and of organic and inorganic materials. Due to its superior spatial resolution, EM has been used extensively although it is not an ideal tool for the investigation of dynamic processes. On the other hand, optical microscopy, especially fluorescence microscopy, offers a non-invasive tool for the investigation of dynamic processes at the microscopic level and so has been one of the essential tools for life science research. However, the spatial resolution of optical microscopy has been limited by the diffraction of light, typically 200–300 nm in the lateral direction in the visible light range. Due to this limitation, optical microscopy has not been applicable to the visualizing of nanometer scale structural dynamics.

This situation has changed drastically in recent years as a result of the development of new optical microscopy techniques the spatial resolution of which goes beyond the diffraction limit (Hell, 2009; Huang et al., 2009, 2010; Patterson et al., 2010; Schermelleh et al., 2010; Gould et al., 2012). These new optical microscopy techniques, referred to as super-resolution (SR) fluorescence microscopy, have been revolutionizing the way in which we investigate the structure and dynamics of a wide variety of nanoscale architectures. The development of these new SR microscopy technologies involves not only the field of optics but also multiple research areas including the development of new fluorophores using organic synthesis or molecular biology technologies; photochemical and photophysical studies on new fluorescent probes to control their optical properties; the development of efficient and high throughput labeling techniques, and the development of new image processing algorithms. Such interdisciplinary research has contributed to the rapid technological evolution of SR fluorescence microscopy.

In this review, I begin with an overview of the current state of SR fluorescence microscopy in nanoscale structural imaging together with recent developments in fluorescent probes. On this basis, I describe quantitative molecular imaging using SR microscopy, which enables the visualizing not only of the nanoscale structures but also dynamic processes at the single-molecule level with diffraction-unlimited spatial resolution. Furthermore, I introduce recent developments of SR microscopy in the field of functional imaging. Using SR microscopy techniques, the physical, chemical, and optical properties of nanoscale architectures can be mapped directly along with their structure. This opens new opportunities for unraveling the functions of a wide range of nanoscale architectures and promises to lead eventually to the development of new (bio-)nanotechnology.

## Principles of Super-Resolution Fluorescence Microscopy

### Diffraction limit and super-resolution

When light emitted by a point source (e.g., a single organic fluorophore) is focused by a lens system, the light rays are not converged to an infinitely small point at the image plane due to the diffraction of light. The width of the spot is approximately 0.6 λ/NA, where λ and NA denote the wavelength of the light and numerical aperture of the optical system (i.e., microscope objective lens), respectively. In the visible wavelengths, the spot size is typically 200–300 nm when a high NA objective lens (NA > 1) is used for imaging. The spot size along the axial axis is typically ~550 nm. The intensity profile of the focused spot is called the point spread function (PSF).

For many decades, the spatial resolution of optical microscopy was improved by decreasing the size of the PSF. Beginning with confocal and multiphoton fluorescence microscopy, which improves the axial resolution slightly, a variety of high-resolution optical microscopy methods have been developed. In 4Pi (Hell and Stelzer, 1992) and I^5^M microscopy (Gustafsson et al., 1999), the spatial resolution has been improved by increasing the NA of the excitation and detection optical system using two opposing objective lenses. Scanning near-field optical microscopy (SNOM) improves the spatial resolution using non-propagating light, an evanescent wave, for excitation as well as detection of the fluorescence signal (Betzig and Trautman, 1992; Johnson et al., 2012). The size of the aperture used for excitation is the key factor, which determines the spatial resolution of SNOM. Although SNOM provides superior lateral resolution, typically an order of magnitude higher than that of normal optical microscopy, the method is applicable only for the study of the surface of a 3D specimen. Metamaterials-based superlens and hyperlens have also been developed, both of which offer sub-diffraction-limited imaging (Fang et al., 2005; Liu et al., 2007; Rho et al., 2010).

### STED microscopy and related techniques

**Figure 1:**
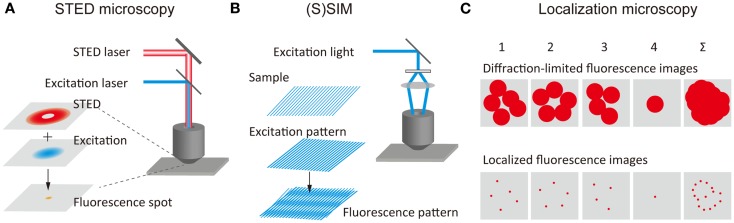
**Principles of super-resolution fluorescence structural imaging**. Schematic illustrations of the principles of **(A)** STED microscopy, **(B)** (S)SIM, and **(C)** localization microscopy.

The concept of stimulated-emission depletion (STED) microscopy was proposed by Stefan Hell (Hell and Wichmann, 1994) and was later demonstrated experimentally (Klar et al., 2000). In STED microscopy, spontaneous fluorescence emission caused by the excitation of the fluorophores by an excitation laser with diffraction-limited spot size is suppressed by a second laser beam (STED beam), which depletes the excited-state through stimulated-emission before the fluorophores emit light through spontaneous fluorescence (Figure 1A). The STED beam with a doughnut-like spatial pattern leads to the depletion of the spontaneous fluorescence at the periphery of the fluorescence spot. When the fluorescence is depleted by an intense STED beam, the fluorescence depletion in the peripheral region is saturated (i.e., saturation of stimulated-emission), resulting in a very small fluorescence spot at the region around the focal point. This corresponds to the reduction of the PSF. An SR fluorescence image can be obtained by scanning the spatially overlapped excitation and STED beams across the sample. Analogous methods such as ground-state depletion (GSD) microscopy (Bretschneider et al., 2007) have been developed using different mechanisms of fluorescence depletion. Collectively, these methods are referred to as reversible saturable optical fluorescence transitions (RESOLFT) microscopy (Hofmann et al., 2005). A spatial resolution of approximately 30 nm in both the lateral and axial directions has been achieved using a STED beam of a 3D doughnut-like spatial pattern (Schmidt et al., 2008). STED microscopy with an imaging speed similar to confocal microcopy (i.e., video-rate) has been reported (Westphal et al., 2008). A rapid RESOLFT microscopy imaging (120 μm × 100 μm-sized fields of view in <1 s) has also been achieved by parallelizing scanning with more than 100,000 beams (Chmyrov et al., 2013). Furthermore, the spatial resolution has been improved by the time-gated detection of fluorescence (Vicidomini et al., 2011), which allows the visualizing of finer structures at relatively low intensities of the STED beam. STED microscopy has also been combined with two-photon excitation microscopy (Bianchini et al., 2012).

### (S)SIM

(Saturated) structured-illumination microscopy [(S)SIM] has been developed by Gustafsson (2005) and Heintzmann et al. (2002). In (S)SIM, samples are illuminated by an excitation light, which has a periodical spatial pattern, typically a sinusoidal pattern (Figure 1B). A fluorescence image recorded under this condition displays low frequency Moiré fringes due to the frequency mixing of the sub-diffraction-limit sample structure and the patterned excitation light; this can readily be resolved by conventional optical microscopy. A high-resolution image is reconstructed by recording multiple images with different phases and orientations of the patterned excitation light (Heintzmann and Gustafsson, 2009). As in the case of STED microscopy, higher spatial resolution can be achieved by illuminating the sample with intense excitation light. The saturation of the fluorescence signal under the intense light illumination introduces a high-frequency component to the spatial pattern of the excitation light, leading to diffraction-unlimited higher spatial resolution. A lateral resolution of better than 50 nm has been achieved using SSIM (Gustafsson, 2005). 3D SIM has also been developed, which uses an excitation light with a 3D periodical pattern (Schermelleh et al., 2008; Shao et al., 2011; York et al., 2013).

### Localization microscopy

The concept of SR localization microscopy was conceived and implemented independently by Eric Betzig (Betzig et al., 2006), Samuel Hess (Hess et al., 2006), and Xiaowei Zhuang (Rust et al., 2006). While most of the SR microscopy methods realize a higher spatial resolution by reducing the size of PSF, these researchers achieved SR by determining extremely precisely the spatial locations of individual molecules. Although the fluorescence spot obtained from a single-molecule is 200–300 nm in size, this being determined by the PSF of the optical system, the spatial location of the molecule can be determined very precisely, localization precision of typically 1–10 nm, by fitting the intensity profile using a Gaussian function (Yildiz et al., 2003; Pertsinidis et al., 2010). In conventional fluorescence microscopy, the fluorescence signal from the entire complement of molecules in the sample is detected simultaneously, and the fluorescence from individual molecules overlaps spatially. Thus, the spatial location of individual molecules cannot be determined precisely. In localization microscopy, at one time only a small fraction of the fluorescent molecules in the sample show fluorescence (i.e., those in the active state), this enabling determination of the precise position of these molecules (Figure 1C). These molecules are then deactivated, and another subset of the fluorescent molecules is then activated and localized precisely. An SR image is reconstructed by mapping the spatial locations of a large number of molecules.

In localization microscopy, fluorescent probe molecules have to be temporally switched between a fluorescent state and a dark sate. The development of photoswitchable fluorescent probe molecules [including, e.g., photoswitchable proteins (Patterson and Lippincott-Schwartz, 2002; Wiedenmann et al., 2004; Habuchi et al., 2005), and photoswitchable organic dyes (Bates et al., 2005; Heilemann et al., 2005)] brought the concept of localization microscopy into reality. These methods have been called (F)PALM [(fluorescence) photoactivated localization microscopy] (Betzig et al., 2006; Hess et al., 2006; Flors et al., 2007) or STORM (stochastic optical reconstruction microscopy) (Rust et al., 2006). Based on a similar concept, other localization microscopy techniques such as dSTORM (direct STORM) (Heilemann et al., 2008), PALMIRA (PALM with independently running acquisition) (Egner et al., 2007), GSDIM (GSD followed by individual molecular return) (Folling et al., 2008), Blink microscopy (Steinhauer et al., 2008), PAINT (point accumulation for imaging in nanoscale topography) (Sharonov and Hochstrasser, 2006) have been developed, and these methods are collectively referred to as SR localization microscopy (Patterson et al., 2010).

The spatial resolution of localization microscopy is determined primarily by the localization precision of individual fluorescent probe molecules. A higher level of precision is achieved through collection of a larger number of photons from individual molecules (Thompson et al., 2002). In addition, the density of the localized molecules significantly affects the spatial resolution of the reconstructed image (van de Linde et al., 2010). According to the Nyquist criterion, the sampling density should be at least twice the desired resolution (Biteen et al., 2008). Therefore, the dense labeling with the fluorophores is essential for successful SR localization microscopy imaging experiments. A lateral resolution of 10–20 nm has been achieved. 3D localization microscopy requires the precise localization of the 3D position of individual molecules. This has been achieved using, for example, optical astigmatism (Huang et al., 2008); double-helix PSF (Pavani et al., 2009); phase-based interferometry with two opposing objective lenses (Shtengel et al., 2009); and double-plane detection of the fluorescence signal (Juette et al., 2008). Using these methods, an axial spatial resolution of 10–75 nm has been achieved. 3D localization microscopy of live cells at a time resolution 1–2 s has been reported (Jones et al., 2011). Confined activation of fluorophores using two-photon (York et al., 2011) as well as selective plane illumination (Zanacchi et al., 2011) has further extended the range of applications of localization microscopy to thick 3D samples.

The concept of localization microscopy has been extended to the reconstruction of a SR image based on the time-dependent fluorescence intensity changes at the ensemble level due to photobleaching and blinking of the fluorophores (Burnette et al., 2011; Simonson et al., 2011; Cox et al., 2012). The statistical analysis of the ensemble level fluctuation of the fluorescence intensity has also been used to reconstruct a SR image without the localization (Dertinger et al., 2009; Dedecker et al., 2012).

A variety of algorithms for the precise and efficient localization of individual fluorescence spots have been proposed for the efficient reconstruction of a high quality SR image (Mortensen et al., 2010; Holden et al., 2011). These image processing algorithms allow the real-time reconstruction of SR images (Smith et al., 2010; Wolter et al., 2010, 2012).

## Fluorescent Probes and Labeling Techniques for Super-Resolution Microscopy

The SR fluorescence microscopy techniques described above rely on the photophysical and/or photochemical properties of fluorescent probe molecules (e.g., saturation of fluorescence intensity, saturation of stimulated-emission, and the switching between different fluorescent states). New fluorescent probes optimized for SR microscopy have been the subject of intense investigation (Fernandez-Suarez and Ting, 2008).

In STED microscopy, the photostability of the fluorophore is one of the most important factors as the sample is exposed to intense laser illumination. Photobleaching of organic fluorophores occurs frequently via a higher excited-state (Donnert et al., 2006). This is particularly important in STED microscopy as the intense laser illumination could lead to an efficient excitation to the higher excited-state. Organic fluorophores suitable for STED microscopy have been identified by analyzing the energy level of the higher excited-state of the fluorophore (Hotta et al., 2010). Bright and photostable organic fluorophores with appropriate Stokes shifts have been designed for multicolor STED microscopy (Kolmakov et al., 2012; Schill et al., 2013). The photostability of the fluorophores is similarly one of the critical factors in SSIM.

As evident from the principle underpinning the imaging technique, controlling the fluorescent on- and off-states of the fluorophores is critical to making an accurate localization measurement. In the early stage of development of localization microscopy, the temporal control of the fluorescent states was based on the wavelength of fluorescence (van Oijen et al., 1998), photobleaching of the fluorophores (Gordon et al., 2004), or fluorescence blinking of quantum dots (Lidke et al., 2005). However, due to relatively inefficient fluorescent switching behaviors, these approaches did not achieve reconstruction of a SR image.

**Figure 2:**
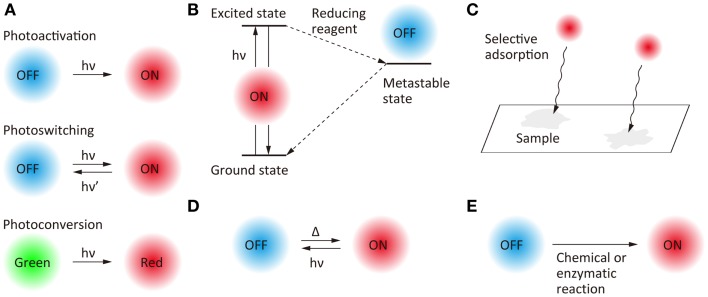
**Scheme of the switching of fluorescence states for super-resolution fluorescence localization microscopy**. **(A)** Photo-induced switching, **(B)** photo-induced redox switching, **(C)** selective adsorption, **(D)** photo- and thermal-induced switching, and **(E)** chemical or enzymatic reaction-induced switching.

Temporal control of the fluorescent state has become possible with the development of photoswitchable molecules (Figure 2A). Photoactivation (i.e., switching from an off-state to an on-state upon illumination at a specific wavelength), photoswitching (i.e., reversible switching between an on-state and an off-state upon illumination at two different wavelengths), and photoconversion (i.e., conversion of fluorescent state from one color to another upon illumination at a specific wavelength) have been the most widely used strategies for the temporal control of the fluorescent states of the probe molecules. Fluorescent proteins for which the fluorescent properties can be switched by light illumination have been studied extensively (Nienhaus and Nienhaus, 2014), these proteins having been designed originally for fluorescence-based optical highlighting (Patterson, 2011). A variety of photoswitchable proteins (Ando et al., 2004; Habuchi et al., 2005; Egner et al., 2007; Flors et al., 2007; Andresen et al., 2008; Stiel et al., 2008; Brakemann et al., 2011), photoactivatable proteins (Patterson and Lippincott-Schwartz, 2002; Subach et al., 2009, 2010; Gunewardene et al., 2011), and photoconvertable proteins (Ando et al., 2002; Wiedenmann et al., 2004; Habuchi et al., 2008; McKinney et al., 2009; Subach et al., 2011; McEvoy et al., 2012; Moeyaert et al., 2014) have been designed for the localization microscopy. Fluorescent proteins, which show both photoactivation and photoconversion (Fuchs et al., 2010), such as Iris FP (Adam et al., 2008) and NijiFP (Adam et al., 2011), have also been designed for SR pulse-chase imaging. Photochromic dyes are well-known as photoswitchable molecules (Irie et al., 2002), and have been used as fluorescent probes for localization microscopy (Folling et al., 2007).

Another approach to the temporal switching of the fluorescence state is to use the excited-state photophysical and photochemical properties of organic fluorophores (Figure 2B). Upon illumination with an excitation light in the presence of appropriate reducing reagents, some organic fluorophores are trapped in a metastable dark state which can act as the off-state of fluorescence. Based on this mechanism, the fluorescent state of Cy dyes, Alexa dyes, and Atto dyes can be switched reversibly between on- and off-states (Bates et al., 2005; Heilemann et al., 2005, 2009; Steinhauer et al., 2008; Vogelsang et al., 2009; Dempsey et al., 2011). Temporal switching of fluorescence has also been achieved by using random adsorption of the fluorophores to the sample (Figure 2C). A combination of random adsorption and photochemical redox switching can be used for a scheme of SR localization microscopy (Yabiku et al., 2013). It is also possible to thermally switch the fluorescence state of some fluorophores from off to on (Figure 2D).

Furthermore, blinking (Watanabe et al., 2010) and photo-induced color changes (Hoyer et al., 2011) of quantum dots, and nanoparticles doped with photochromic dyes (Tian et al., 2011) have been reported as fluorescent probes for SR localization microscopy. Recently, the switching of fluorescent states without the requirement for light illumination has been achieved by chemical (Schwering et al., 2011; Vaughan et al., 2012) and enzymatic (Lee et al., 2013) reactions (Figure 2E).

Specific labeling of the target molecules at an appropriate spatial density determined by the Nyquist criterion is one of the factors, which affects the quality of SR microscopy experiments (Lukinavicius et al., 2013). Immunostaining (Huang et al., 2008) and genetic fusion to fluorescent proteins (Betzig et al., 2006) are the most frequently used approaches for SR fluorescence imaging of biological samples. Fluorophores targeting specific intracellular components such as DNA (Flors et al., 2009; Benke and Manley, 2012) and membranes (Shim et al., 2012) have also been used for SR fluorescence imaging. Hybrid systems of genetic tagging and external fluorophores, such as SNAP-tag, TMP-tag, CLIP-tag, and Halo-tag, provide an alternative tool for specific labeling of the target (Hein et al., 2010; Lee et al., 2010; Wombacher et al., 2010; Wilmes et al., 2012; Stagge et al., 2013). Using these systems, a wide variety of external fluorophores can be attached to the non-fluorescent genetic tags, enabling the use of specific fluorophores, which possess the particular fluorescent properties required for an imaging experiments.

## Nanoscale Structural Imaging

### Life science

Research in the life sciences field probably benefits most from the development of SR fluorescence microscopy techniques. These techniques are becoming an essential tool for investigating nanoscale architectures in fixed as well as in live cells. Subcellular organelles typically have structural dimensions of tens to hundreds of nanometers, which can be resolved readily by SR microscopy. The structure and protein organization in cellular components, such as mitochondrial cristae (Schmidt et al., 2009) and nucleoids (Kukat et al., 2011), the nuclear pore complex (Schermelleh et al., 2008), and focal adhesions (Kanchanawong et al., 2010; Rossier et al., 2012), have been visualized directly. In addition, the structure and dynamics of intracellular molecular assembly (e.g., clusters of membrane proteins) have been the subject of intense study using SR microscopy techniques (Hess et al., 2007; Sieber et al., 2007; Eggeling et al., 2009; Mueller et al., 2011; van den Bogaart et al., 2011; Wurm et al., 2011; Itano et al., 2012). In microbiology, SR microscopy has been a powerful tool for studying the spatiotemporal behaviors of protein complexes in bacterial cells, such as the mechanism of spatial organization of structural proteins (Biteen et al., 2008; Ptacin et al., 2010; Buss et al., 2013), and nucleoid-associated proteins (Lee et al., 2011). In neuroscience, SR microscopy has revealed the structure and dynamics of synaptic vesicles (Willig et al., 2006; Westphal et al., 2008; Hua et al., 2011), dendritic spines (Nagerl et al., 2008), and axons (Xu et al., 2013). SR microscopy has also been applied to the study of protein redistribution in the human immunodeficiency virus (HIV-1) (Chojnacki et al., 2012), the interaction between HIV-1 and a transmembrane protein on a host cell (Lehmann et al., 2011), the structure of telomeres (Doksani et al., 2013), the nanoscale architecture of chromatin (Ribeiro et al., 2010), and the mapping of specific sequences of DNA (Neely et al., 2010).

### Materials science

In the field of materials science, while SR microscopy has just come to be recognized as an effective research tool, it has already been applied for studying the architecture of nanomaterials. 3D nanostructures and morphology in the range of tens to hundreds of nanometers, such as colloidal crystals (Harke et al., 2008), block copolymers (Ullal et al., 2009), graphene (Stoehr et al., 2012), and DNA barcodes (Lin et al., 2012a) and origami (Jungmann et al., 2010) have been visualized directly by SR microscopy. Also, the nanoscopic spatial distribution of fluorescent nitrogen vacancies in diamond (Rittweger et al., 2009) and lipid bilayer phases (Kuo and Hochstrasser, 2011) has been mapped using SR microscopy.

## Nanoscale Quantitative Molecular Imaging

Super-resolution localization microscopy is based on the fluorescence imaging of individual molecules. Using this method, SR images are reconstructed through the spatial localization of individual molecules by spatiotemporally controlling the activation and deactivation of the fluorescence signal. This experimental scheme provides a unique opportunity to investigate the spatiotemporal dynamics of single-molecules along with the diffraction-unlimited structural imaging of nanoscale architectures.

**Figure 3:**
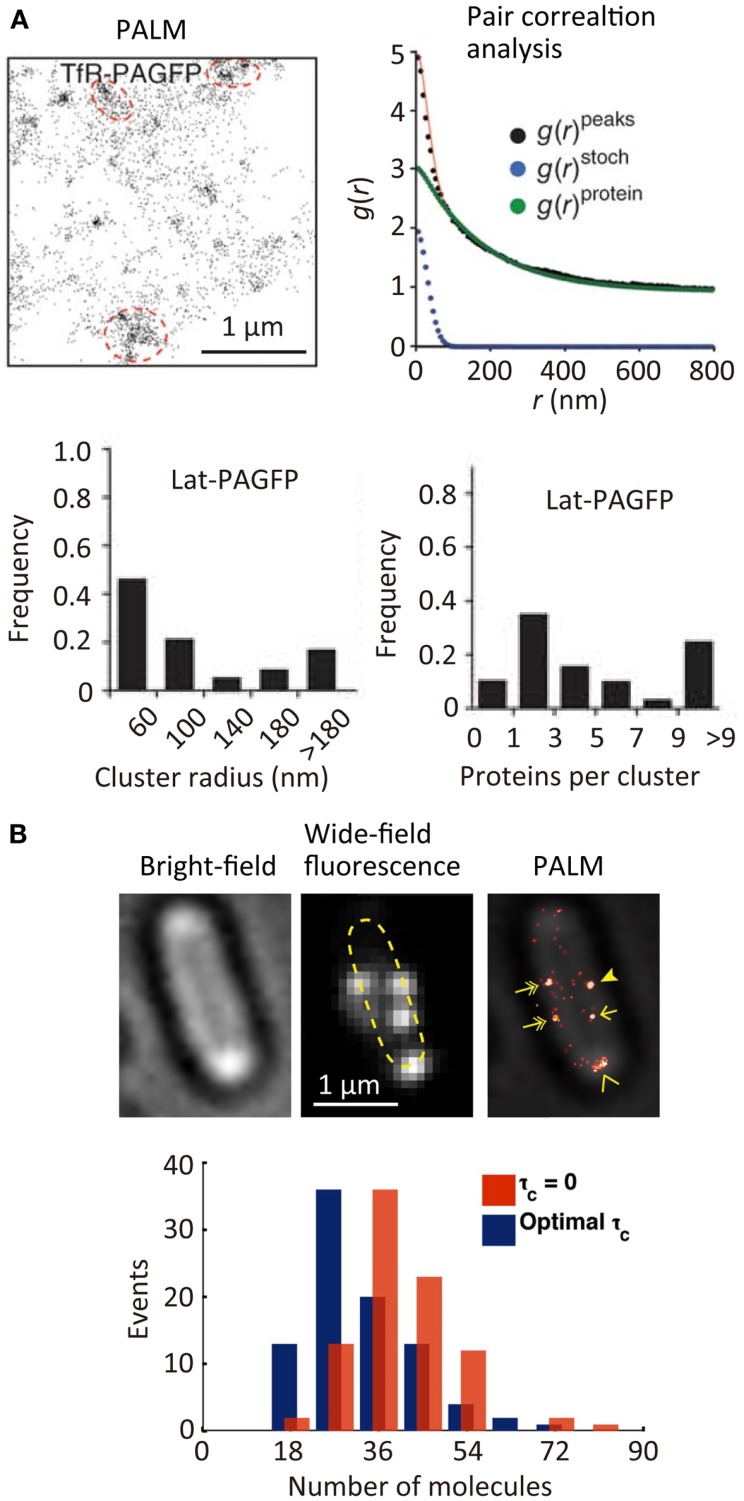
**Quantitative super-resolution fluorescence imaging**. **(A)** (Top left) PALM image of transferrin receptor (TfR) across the plasma membrane of COS7 cells. A photoactivatable fluorescent protein PAGFP is fused to TfR; (top right) plot of calculated autocorrelation function [*g*(*r*)^peaks^] of PAGFP molecules in the PALM image. *g*(*r*)^stoch^ and *g*(*r*)^protein^ are the correlation owing to multiple appearances of a single protein and the protein correlation. The cluster size and average numbers of the molecule in clusters are estimated by a fitting of the autocorrelation plot with an exponential decaying function; (bottom) size and number of molecules in clusters of a transmembrane protein, Lat (Sengupta et al., 2011). **(B)** (Top) bright-field microscopy, wide-field fluorescence microscopy, and PALM images of a flagellar motor protein, FliM, in bacterial cells. A photoconvertable fluorescent protein Dendra2 is fused to FliM; (bottom) frequency histogram of number of FliM molecules in each cluster determined using the Fermi activation with optimal tolerance time (blue bars). The red bars show the histogram without optimizing the tolerance time (Lee et al., 2012). Reproduced with permission from Sengupta et al. (2011), copyright 2011, Nature Publishing Group **(A)**, and Lee et al. (2012), copyright 2012, National Academy of Science, USA **(B)**.

The spatial distribution of membrane proteins is believed to play an important role in many ligand–receptor binding systems, such as cell adhesion. Since the spatial locations of individual molecules can be determined with a precision of approximately 10 nm in SR localization microscopy, the spatial distribution of individual molecules [e.g., transferrin receptor (TfR) across the plasma membrane, see Figure 3A (Sengupta et al., 2011)] can be characterized quantitatively using statistical analysis (Owen et al., 2010; Scarselli et al., 2012; Veatch et al., 2012; Malkusch et al., 2013). The size of the TfR cluster in the membrane (~150 nm in radius) is estimated roughly from the autocorrelation function [g(*r*)^protein^] obtained by pair correlation analysis of the spatial distribution of the individual molecules. This analysis allows a quantitative estimation of the size of molecular clusters together with the average number of molecules in a cluster (Sengupta et al., 2011). Although in principle the quantitative determination of the number of molecules in a cluster is possible, in practice this is not straightforward due to the blinking behavior of most of the fluorophores. To achieve such quantitative determination, a single-molecule counting method has been developed, which enables determination of the number of the molecules within diffraction-limited space with high accuracy (Lee et al., 2012). In this method, the counting error is minimized by temporally modulating the intensity of the activation laser (i.e., Fermi activation), which maximally separates the activation of the different molecules together with optimizing the tolerance time *τ*_c_ which is the characteristic time of the blinking. Using this approach, the number of photoconvertable proteins, which are fused to a flagellar motor protein, FliM, has been determined accurately along with obtaining an SR image of FliM in the cells (Figure 3B) (Lee et al., 2012). The development of less blinking fluorophores will further improve the accuracy of counting the number of the molecules.

**Figure 4:**
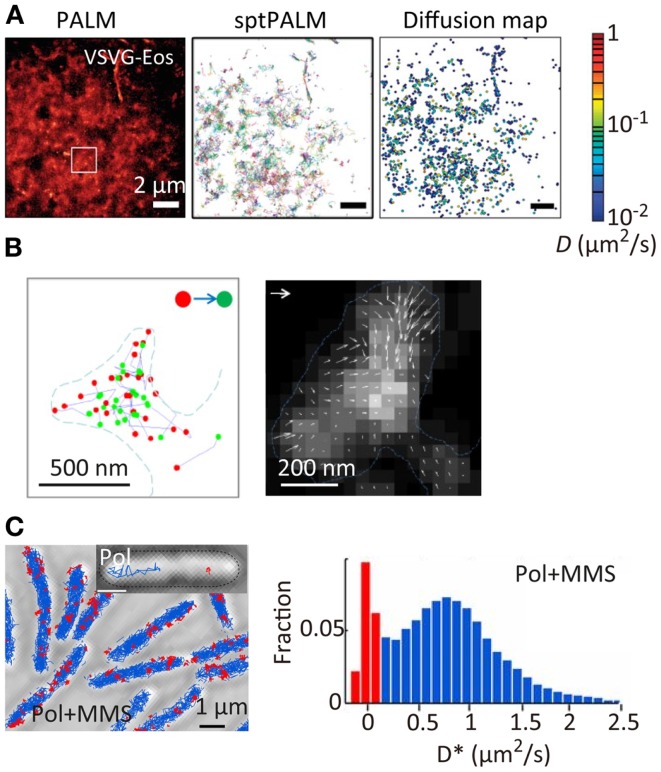
**Super-resolution fluorescence molecular imaging**. **(A)** PALM image, sptPALM image, and diffusion map of a membrane protein, VSVG, in COS7 cells. A photoconvertable fluorescent protein tdEosFP is fused to VSVG. The PALM image was obtained by the temporal activation of tdEosFP–VSVG followed by the localization and reconstruction of the image. The sptPALM image was obtained by tracking the activated tdEosFP–VSVG molecules. The diffusion map was obtained by calculating the diffusion coefficient of individual tdEosFP–VSVG molecules using the diffusion trajectories (Manley et al., 2008). **(B)** (Left) the first (red) and last (green) localized positions of single actin molecules within dendritic spines. The positions were determined by the sptPALM imaging. A photoactivatable fluorescent protein PAGFP is fused to actin; (right) actin dynamics within individual spines. Orientation and length of the arrows represent direction and velocity of actin flow. They were calculated using the first and last positions of the diffusion trajectories within the radius of 1 camera pixel (111 nm). Gray scale represents molecular density. Vector = 100 nm/s (Frost et al., 2010). **(C)** (Left) sptPALM image of a DNA-binding protein, DNA polymerase I (Pol), in *E. coli* cells in the presence of methyl methanesulfonate (MMS). A photoactivatable fluorescent protein PAmCherry is fused to Pol. Inset shows examples of tracks of diffusing Pol (blue) and bound Pol (red); (right) distribution of diffusion coefficient for Pol under constant MMS treatment (Uphoff et al., 2013). Reproduced with permission from Manley et al. (2008), copyright 2008, Nature Publishing Group **(A)**, Frost et al. (2010), copyright 2010, Elsevier **(B)**, and Uphoff et al. (2013), copyright 2013, National Academy of Science, USA **(C)**.

Super-resolution localization microscopy has been further combined with single-particle tracking (SPT). SPT is a powerful tool for characterizing the spatiotemporal behavior of individual molecules, especially the diffusional motion of molecules in nanoscopic heterogeneous structures such as cell membranes (Douglass and Vale, 2005) and microporous materials (Kirstein et al., 2007). However, the method can be applied only to relatively low density mapping of the diffusional behavior of individual molecules. This makes it difficult to connect directly the spatiotemporal behavior of the molecules to the nanoscopic structure of the sample. The combination of SPT and SR localization microscopy allows high-density spatial mapping of the diffusional behavior of individual molecules through tracking single fluorescent probe molecules activated in a temporally controlled manner (Figure 4A) (Manley et al., 2008; Giannone et al., 2010; Rossier et al., 2012). This method has been applied to the study of actin molecule dynamics within dendritic spines. While actin plays numerous roles in synaptic transmission, its spatiotemporal behavior has not been well-characterized due to the small (submicrometer) size of the spines. Using this method, the highly heterogeneous velocity of individual actin molecules within the spines has been demonstrated (Figure 4B) (Frost et al., 2010). The same approach has been applied to the study of DNA repair in bacterial cells. When DNA is damaged in a cell, a freely diffusing DNA polymerase (Pol) binds to the damaged site. The spatiotemporal dynamics of DNA repair events have been mapped through high-density tracking of the fluorescently labeled DNA Pol (Figure 4C) (Uphoff et al., 2013). As is evident from these studies, biological processes are precisely regulated by the spatial and temporal dynamics of protein molecules. The tools for quantitative molecular imaging such as cluster analysis, single-molecule counting, and SPT SR microscopy will provide new opportunities to investigate complex biological processes at the molecular level.

## Super-Resolution Functional Imaging

Super-resolution fluorescence microscopy has been developed in an attempt to visualize nanoscale structures and their dynamics directly. Recently, these techniques have become recognized as an effective means to analyze nanoscale physical, chemical, and optical properties.

### Catalytic reactions

**Figure 5:**
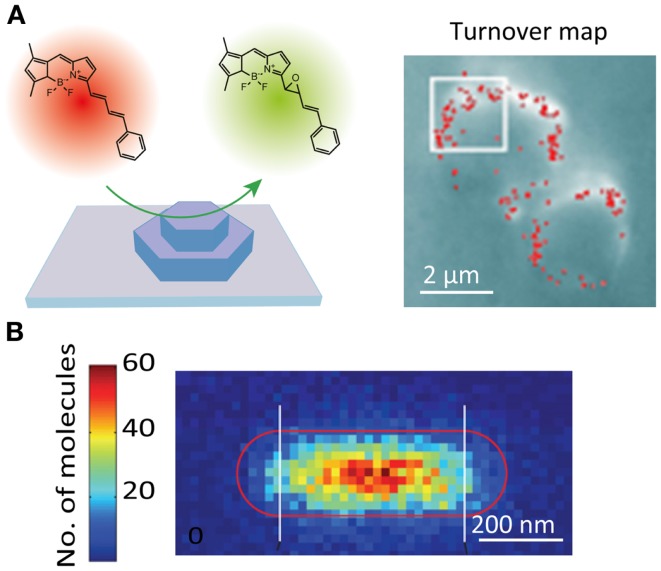
**Super-resolution fluorescence imaging of catalytic reactions**. **(A)** (Left) schematic illustration of the strategy for monitoring individual epoxidation reaction events catalyzed by mesoporous titanosilicates, Ti-MCM-41. The active sites are fluorescently visualized and localized by the catalytic reaction-induced color conversion of the fluorophore; (right) localization microscopy image of individual turnover. The red dots show positions of fluorescent spots originating from individual product molecules (De Cremer et al., 2010). **(B)** Super-resolution imaging of deacetylation reaction catalyzed by Au@mSiO_2_ nanorod. The positions of the reaction product on the nanorod are visualized by localization microscopy and plotted in the 2D histogram, which shows the position-dependent catalytic activity (Zhou et al., 2012). Reproduced with permission from De Cremer et al. (2010), copyright 2010, Wiley **(A)**, and Zhou et al. (2012), copyright 2012, Nature Publishing Group **(B)**.

The catalytic activity of solid catalysts is governed by their nanoscale structural heterogeneities. However, a lack of appropriate methodology had hampered attempts to connect the activity with the nanoscale structure of the catalysts. Recent studies have demonstrated that the active sites of catalytic reactions can be visualized at a spatial resolution of tens of nanometers using SR localization microscopy (Roeffaers et al., 2007). In these studies, the spatial locations of the active sites are determined by localizing fluorescent molecules generated by the catalytic reaction (Figure 5A). The SR catalytic activity imaging revealed that mesoporous particles such as zeolite (Roeffaers et al., 2009) and titanosilicate Ti-MCM-41 (De Cremer et al., 2010) show catalytic activity only at the surface of the particles due to the limited access of the substrate molecules to the catalytic sites inside the particles (Figure 5A). Heterogeneous catalytic activity on the surface of the gold nanorod catalyst has also been reported (Figure 5B) (Zhou et al., 2012; Andoy et al., 2013). The catalytic activity of carbon nanotubes (Xu et al., 2009) and titanium dioxide nanoparticles (Tachikawa et al., 2013) has also been characterized by SR fluorescence microscopy.

### Optical properties

**Figure 6:**
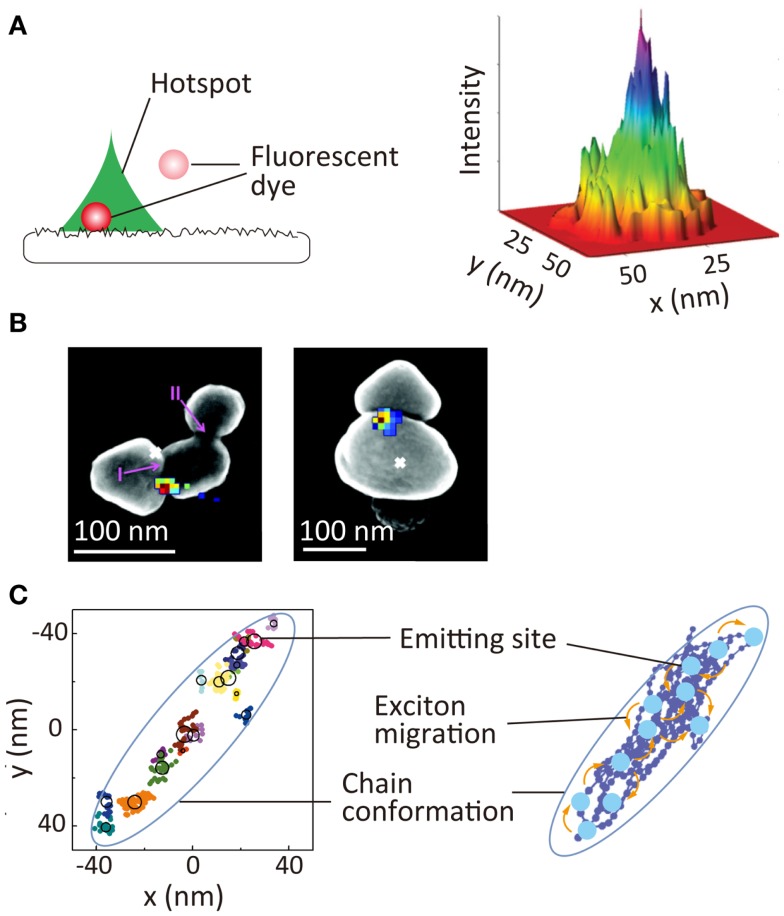
**Super-resolution imaging of optical properties of nanoscale architectures**. **(A)** Super-resolution imaging of local electromagnetic field enhancement. (Left) principle of Brownian motion super-resolution imaging; (right) super-resolution image of a hotspot on the surface of an aluminum film (Cang et al., 2011). **(B)** Super-resolution image of SERS signal of rhodamine 6G dyes adsorbed at gap between two silver nanoparticles. Overlay of the SERS spatial intensity map (colored pixels) and luminescent centroid (white ×) with an SEM image of silver nanoparticle aggregate (Weber et al., 2012). **(C)** (Left) schematic illustration nanoscale photophysical processes occurring in a single conjugated polymer molecule. (Right) 2D spatial map of emitting sites within a single conjugate polymer chain; reproduced with permission from Cang et al. (2011), copyright 2011, Nature Publishing Group **(A)**, Weber et al. (2012), copyright 2012, American Chemical Society **(B)**, Habuchi et al. (2011), copyright 2011, PCCP Owner Societies **(C)**.

An electromagnetic field near metallic nanostructures is enhanced significantly through localized surface plasmon resonance. This phenomenon has been used extensively for chemical and biological sensing as well as for the development of plasmonic optics. While plasmonic hotspots, such as nanoscale gaps and protrusions, are responsible for the enhancement of the local electromagnetic field, it has been difficult to visualize these hotspots directly because nanometer scale spatial resolution is required. SR localization microscopy offers the unique possibility of visualizing a hotspot through measuring the hotspot-induced fluorescence intensity enhancement (Cang et al., 2011; Lin et al., 2012b; Wei et al., 2013). In these studies, the enhancement of the local electromagnetic field was quantified by a precise localization of the positions of freely diffusing fluorophores near the hotspots along with the quantitative analysis of the fluorescence intensity of these fluorophores. This allows visualization of the size and shape of the hotspots, as well as the electromagnetic field enhancement within the single hotspot (Figure 6A). Hotspots on an aluminum film have been visualized using this method (Figure 6A) (Cang et al., 2011). A similar approach has been used to visualize hotspots in the gap regions between silver nanoparticle aggregates (Figure 6B). In this study, surface-enhanced Raman scattering (SERS) signals of adsorbed organic dye molecules were used to visualize the hotspots (colored pixels in Figure 6B) (Weber et al., 2012). The spatial locations of the hotspots showed a deviation from the luminescence center of the aggregates, which clearly demonstrated the gap-induced enhancement of the electromagnetic field.

Super-resolution localization microscopy is also a powerful tool for visualizing nanoscale photophysical processes occurring within single conjugated polymer molecules, these comprising an important class of material for optoelectronic applications, such as solar cells and light emitting diodes. Conjugated polymers have also been used as biological sensors and fluorescent markers. Exciton migration through energy transfer within the molecules in the excited-state is one of the critical factors for these applications. Typically, conjugated polymer molecules have a structural dimension of tens of nanometers, and the fluorescence is emitted from a localized region within the molecule, the emitting site, due to the exciton migration along the chain. The direct visualization of exciton migration requires visualization of the spatial locations of the emitting sites. Using SR localization microscopy, the spatial locations of the emitting sites within the single chain have been mapped (Figure 6C) (Habuchi et al., 2009, 2011); this has provided new insights into the exciton migration occurring within the conjugated polymer molecules and the relationship of this migration with the conformational state of the molecules (Vacha and Habuchi, 2010; Bolinger et al., 2011).

### Nanoscale fluid dynamics

**Figure 7:**
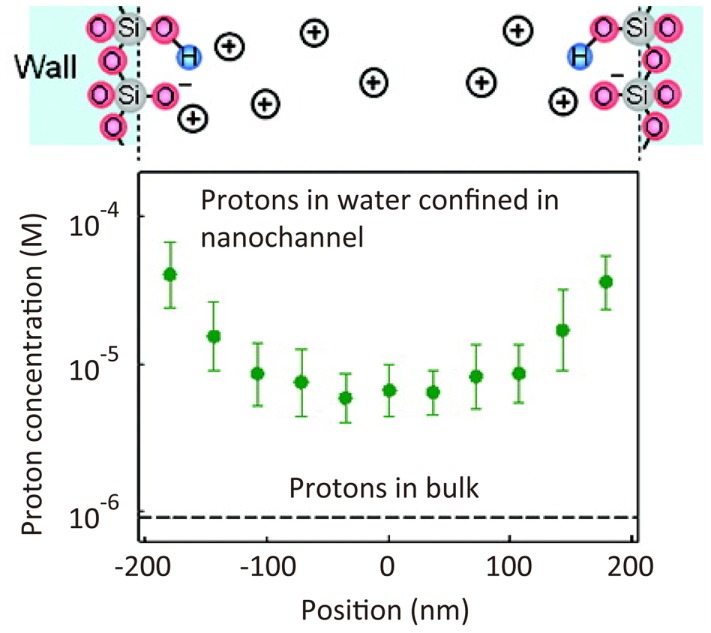
**Super-resolution imaging of proton concentration in a nanochannel**. pH profile in a 410 nm width nanochannel determined by the spatial intensity map of fluorescence intensity of fluorescein dye (Kazoe et al., 2011). Reproduced with permission from Kazoe et al. (2011), copyright 2011, American Chemical Society.

Micro- and nano-fluidics are key technologies in the field of broad analytical science. The properties of fluids in a submicrometer-size nanochannel are expected to be dramatically different from those in a bulk flow. However, it has been difficult to measure such fluid properties experimentally because nanoscale spatial resolution is required. Using STED microscopy, a pH profile has been determined for a nanochannel on the basis of a spatial intensity map of the fluorescence intensity of fluorescein dye, the intensity of which varies with solution pH (Figure 7) (Kazoe et al., 2011).

## Conclusion and Outlook

The principles and applications of SR fluorescence microscopy techniques have been described in this review. From a technological point of view, both the spatial and time resolution of SR fluorescence microscopy have been improved significantly over the past few years. Indeed, current SR fluorescence microscopy allows visualization of the molecular organization of large protein assemblies (Loeschberger et al., 2012; Szymborska et al., 2013). Furthermore, recent efforts toward a greater time resolution allow dynamic processes to be visualized in an almost real-time way. The development of fluorophores and optical systems, which enable to collect larger number of photons within a sufficiently short time will further improve the temporal resolution. It is anticipated that optical imaging of dynamic processes with a spatial resolution at the molecular level will soon be possible. From a scientific perspective, SR fluorescence microscopy offers an invaluable opportunity to unravel complex biological processes at the molecular level, and is likely to soon become an essential method in life science research. Currently, SR fluorescence microscopy is considered to be complementary to EM in the field of materials science; further improvement of time resolution, however, will broaden the use of SR fluorescence microscopy in this area.

Recently, SR fluorescence microscopy has been combined with other techniques such as optical tweezers (Heller et al., 2013), to provide insights into the mechanical properties of individual molecules in nanoscale systems. A combination of SR fluorescence microscopy with the chromosome conformation capture technique (Wang et al., 2011; Larkin et al., 2013) would reveal the mechanisms of spatiotemporal chromosome organization. In addition to nanoscale imaging, SR fluorescence microscopy is also of application in nanoscale optical writing (Grotjohann et al., 2011) and fabrication (Fischer et al., 2010). Imaging of non-fluorescent species (Wang et al., 2013) further expands the applicability of SR fluorescence microscopy. The advance of SR optical microscopy techniques along with nanoscale functional imaging will be an essential part of the development of new (bio-)nanotechnology.

## Conflict of Interest Statement

The author declares that the research was conducted in the absence of any commercial or financial relationships that could be construed as a potential conflict of interest.
